# Bid-Induced Release of AIF/EndoG from Mitochondria Causes Apoptosis of Macrophages during Infection with *Leptospira interrogans*

**DOI:** 10.3389/fcimb.2017.00471

**Published:** 2017-11-14

**Authors:** Wei-Lin Hu, Hai-Yan Dong, Yang Li, David M. Ojcius, Shi-Jun Li, Jie Yan

**Affiliations:** ^1^Department of Medical Microbiology and Parasitology, Zhejiang University School of Medicine, Hangzhou, China; ^2^Division of Basic Medical Microbiology, State Key Laboratory for Diagnosis and Treatment of Infectious Diseases, The First Affiliated Hospital, Zhejiang University School of Medicine, Hangzhou, China; ^3^Department of Medical Microbiology and Immunology, Wenzhou Medical University, Wenzhou, China; ^4^Department of Biomedical Sciences, University of the Pacific, Arthur Dugoni School of Dentistry, San Francisco, CA, United States; ^5^Institute of Communicable Disease Control and Prevention, Guizhou Provincial Centre for Disease Control and Prevention, Guiyang, China

**Keywords:** apoptosis, *Leptospira*, Bid, AIF, EndoG, macrophage

## Abstract

Leptospirosis is a global zoonotic infectious disease caused by pathogenic *Leptospira* species. Leptospire-induced macrophage apoptosis through the Fas/FasL-caspase-8/3 pathway plays an important role in the survival and proliferation of the pathogen in hosts. Although, the release of mitochondrial apoptosis-inducing factor (AIF) and endonuclease G (EndoG) in leptospire-infected macrophages has been described, the mechanisms linking caspase and mitochondrion-related host-cell apoptosis has not been determined. Here, we demonstrated that leptospire-infection induced apoptosis through mitochondrial damages in macrophages. Apoptosis was caused by the mitochondrial release and nuclear translocation of AIF and/or EndoG, leading to nuclear DNA fragmentation. However, the mitochondrion-related CytC-caspase-9/3 pathway was not activated. Next, we found that the release and translocation of AIF and/or EndoG was preceded by the activation of the BH3-interacting domain death agonist (Bid). Furthermore, our data demonstrated that caspase-8 was activated during the infection and caused the activation of Bid. Meanwhile, high reactive oxygen species (ROS) trigged by the infection caused the dephosphorylation of Akt, which also activated Bid. In conclusion, Bid-mediated mitochondrial release of AIF and/or EndoG followed by nuclear translocation is a major mechanism of leptospire- induced apoptosis in macrophages, and this process is modulated by both caspase-8 and ROS-Akt signal pathways.

## Introduction

Pathogenic *Leptospira* species are the causative agents of leptospirosis, a world-wide zoonotic infectious disease (Bharti et al., 2003). When infected with the spirochete after contact with water or soil contaminated with the infected animals' urine, individuals show different clinical symptoms such as fever, myalgia, jaundice, pulmonary diffuse hemorrhage, and kidney injury (McBride et al., 2005; Adler and de la Pena Moctezuma, 2010). However, to date, the mechanisms underlying the pathogenicity of this spirochete remain poorly understood (Palaniappan et al., 2007; Fouts et al., 2016).

Macrophages and neutrophils play important roles in the innate immunity against microbial infections through their ability to phagocytose pathogens. However, in contrast to many other bacterial pathogens, while both of these phagocytic cells are able to phagocytose leptospires, only macrophages are able to kill intracellular leptospires (Davis et al., 2009). Therefore, phagocytosis by macrophages plays a critical role in the defense against leptospiral infection of individuals that lack acquired immunity. On the other hand, the ability to defend against phagocytosis by macrophages contributes to the virulence of *Leptospira* species (Merien et al., 1997; Xue et al., 2010, 2013).

Pathogen-induced macrophage apoptosis is thought to be a common end-point of different pathogenic strategies (Navarre and Zychlinsky, 2000). Certain pathogenic bacteria, viruses, and protozoan parasites stimulate apoptosis of host cells during infection to enhance their survival or parasitism in hosts (Barber, 2001; Luder et al., 2001; Ashida et al., 2011). Merien et al. first reported that the strain Verdun belonging to the serogroup Icterohaemorrhagiae serovar Icterohaemorrhagiae of *Leptospira interrogans* (*L. interrogans*), a major pathogenic *Leptospira* species, causes apoptosis of infected macrophages and Vero cells (Merien et al., 1997). Our previous studies also demonstrated that infection with *L. interrogans* serovar Lai strain Lai induces apoptosis and necrosis in macrophages and fibroblasts (Li et al., 2007). Caspases act as the critical effectors of apoptosis, mainly through membrane Fas/FasL-caspase-8,−3 or the mitochondria-related cytochrome c (CytC)-caspase-9,−3 pathway (Riedl and Shi, 2004). Our previous studies demonstrated that Fas-associated death domain (FADD)-caspase-8,−3-dependent or p53 regulated mitochondrion-related caspase-9-independent pathways were activated during apoptosis of macrophages infected with *L. interrogans* strain Lai (Jin et al., 2009; Hu et al., 2013). However, the mechanisms linking caspase and mitochondrion-related host-cell apoptosis remain unknown.

It is well-established that the mitochondrion is the control center for apoptosis or necrosis (Green and Kroemer, 2004). Our previous study found that leptospire-infected macrophages show visible mitochondrial damage such as swelling and disappearance of cristae (Li et al., 2007), and pretreatment of macrophages with a broad-spectrum caspase inhibitor does not completely block apoptosis (Jin et al., 2009; Hu et al., 2013). Apoptosis inducing factor (AIF) and endonuclease G (EndoG) that are normally located in mitochondria can act as mediators of caspase- independent apoptosis (Li et al., 2001; Cande et al., 2004; Cregan et al., 2004). Our previous study showed that AIF and/or EndoG was released from macrophage mitochondria during a leptospire-infection. However, knockdown or blocking of p53 decreased but did not block the release of AIF and EndoG, and the release of AIF could be observed in p53-deficient human monocyte cells (Hu et al., 2013), suggesting that other factors also mediate the release of AIF and/or EndoG. Therefore, in this study we investigated the signaling pathway of AIF and EndoG resulting in apoptosis of macrophages during infection with *L. interrogans* serovar Lai strain Lai. Furthermore, we determined the roles of BH3 interacting domain death agonist (Bid), which modulates FADD-caspase-dependent and mitochondrion-related caspase-independent apoptotic pathways during apoptosis of leptospire-infected macrophages (Landshamer et al., 2008; Guo et al., 2015). The results presented in this study indentify the activation of Bid-AIF/ EndoG as a novel mechanism by which pathogenic *Leptospira* species induce apoptosis in macrophages during an infection.

## Materials and methods

### Leptospiral strain, cell lines, and culture

*L. interrogans* serogroup Icterohaemorrhagiae serovar Lai strain Lai, the dominant pathogenic strain in China (Hu et al., 2014), was provided by the Chinese National Institute for the Control of Pharmaceutical and Biological Products in Beijing, China, and was cultivated at 28°C in Ellinghausen-McCullough-Johnson-Harris (EMJH) liquid medium (Hu et al., 2013). The cell lines J774A.1 and THP-1 were provided by the Cell Bank in the Institute of Cytobiology, Chinese Academy of Science in Shanghai, China. The cells were cultured in RPMI-1640 medium (Gibco, USA) containing 10% fetal calf serum (FCS, Gibco), 100 U/mL penicillin and 100 μg/mL streptomycin (Sigma, USA) in an atmosphere of 5% CO2 at 37°C. In particular, THP-1 cells should be induced to differentiate into macrophages before use by stimulation with 10 ng/mL phorbol 12-myristate 13-acetate (PMA, Sigma) at 37°C for 24 h (Hu et al., 2013).

### Induction of apoptosis in macrophages by leptospira

Freshly cultured leptospires in EMJH medium was centrifuged at 17,200 × g for 15 min (15°C). After washing twice with autoclaved PBS, the precipitated leptospires were suspended in antibiotic-free RPMI-1640 medium with 2.5% FCS. The leptospires in the suspension were counted under the dark-field microscope with a Petroff-Hausser counting chamber (Fisher Scientific, USA) (Hu et al., 2013). J774A.1 or THP-1 cells were seeded in 6-well or 12-well culture plates (Corning, USA) and incubated for 12-h in an atmosphere of 5% CO_2_ at 37°C. After washing with PBS, the cell monolayers were infected with the spirochete at a multiplicity of infection (MOI) of 10, 50, 100, or 500 leptospires per host cell, and incubated for 1, 2, 4, or 8 h at 37°C.

### Detection of leptospira-induced apoptosis

Annexin V-FITC apoptosis detection kit (BioVision, USA) was used to detect the apoptotic ratios of the leptospire-infected cells. According to the manufacturer's protocol, the harvested cells were resuspended in annexin V binding buffer containing annexin V and PI dyes and incubated in the dark for 15 min at room temperature (Hu et al., 2013). The stained cells were immediately analyzed by using a flow cytometer (FC500 MCL, Beckman) to distinguish the cells in apoptosis (annexin V^+^/PI^−^) from necrosis (annexin V^+^/PI^+^). In this experiment, healthy cells before infection were used as the controls.

### Transmission electron microscopy

The leptospire-infected macrophages were collected by centrifugation at 1,000 × g for 10 min at 4°C, fixed in 2.5% glutaraldehyde at 4°C for 2 h and then washed three times with PBS. The harvested pellets were post-fixed with 1% osmium tetroxide, and then rinsed, dehydrated and embedded in epoxy resin (Sigma). Ultrathin sections on 100–150 mesh nickel grids (Plano, Germany) were stained with lead citrate and uranyl acetate (Jin et al., 2009). Finally, the morphological changes in each of the types of leptospire-infected macrophages were observed under a TECNAI-10 transmission electron microscope (Philips, Holland).

### Detection of mitochondrial transmembrane potential

The changes in mitochondrial membrane potential (MMP) was detected by using JC-1 (Molecular Probes, USA) (St-Louis and Archambault, 2007). In healthy mitochondria with a high MMP JC-1 shows red fluorescence, while the color shifts from red to green when the MMP is lost. Briefly, leptospire-infected macrophages were stained with 10 μg/mL JC-1 at 37°C for 15 min, washed three times with PBS and then observed using a fluorescence microscope (Zeiss, Germany) as well as measured by flow cytometry.

### Blockage and RNA interference test

For blockage test, the cell monolayers were pretreated with 10 μM cyclosporine A (CSA, a blocker of mitochondrial permeability transition (MPT), Sigma) for 30 min, 50 μM Z-VAD-FMK (pan-caspase inhibitor), Z-IETD-FMK (caspase-8 inhibitor), Z-LEHD-FMK (caspase-9 inhitor, Sigma) for 1 h at 37°C (Jin et al., 2009; Hu et al., 2013), 25 μM LY294002 (PI3K/Akt pathway inhibitor, Sigma) for 1 h at 37°C, or 10 mM N-acetyl-cysteine (NAC, an anti-oxidant, Sigma) for 1 h at 37°C. In these tests, macrophages without blocker pretreatment were used as the controls. For RNA interference test, small interfering RNAs (siRNAs) specific to silence the mouse or human *bid, aif*, and *endoG* genes were designed and synthesized by Invitrogen Co. (Shanghai, China). The sequences of siRNAs are 5′-CGACUGUCAACUUUAUUAATT-3′ in J774A.1 and 5′-GGCCACUGUUUGGAAAUAATT-3′ in THP-1 (targeting *bid*); 5′-GGAAAUAUGGGAAAGAUCCTT-3′ in J774A.1 and 5′-GGCUACGUCCAGGAGCGCACC-3′ in THP-1 (targeting *aif*); 5′-GGACCGAGGCTGATGGGAA-3′ in J774A.1 and 5′-AAGAGCCGCGAGUCGUACGUG-3′ in THP-1 (targeting *endoG*). The transfection was performed using Lipofectamine-2000 (Invitrogen), according to the manufacturer's protocol.

### Determination of caspase-8 and−9 activity

The activity of caspase-8 or−9 in the leptospire-infected macrophages was measured using a caspase fluorometric assay kit (BioVision) according to the manufacturer's protocol (Jin et al., 2009). Briefly, the leptospire-infected macrophages were trypsinized and subsequently collected by centrifugation as described above. After washing with cold PBS, the cell pellets were suspended with 50 μL chilled lysis buffer for 10 min on ice. The lysates were clarified by 10,000 × g centrifugation for 10 min at 4°C and the supernatants were collected for determination of protein concentration using a BCA protein quantitation kit (Thermo Scientific, USA). Equal amounts of each of the supernatants with the same protein concentration were incubated with 10 mM dithiothreitol and 50 μM caspase fluorometric substrates (Ac-IETD-AFC for caspase-8 and Ac-LEHD-AFC for caspase-9) at 37°C for 1 h (Jin et al., 2009). Fluorescence intensity changes due to the release of AFC (reflecting caspase activity) were measured using a microplate reader (Bio-Rad, Hercules, CA).

### Western blot assay

Fractions of mitochondria and cytosol from the leptospire-infected J774A.1 or THP-1 cells (MOI 100) were prepared using a mitochondria/cytosol fractionation kit (BioVision) following the manufacturer's protocol. Equivalent amounts of protein were separated by sodium dodecyl sulfate polyacrylamide gel electrophoresis (SDS-PAGE) and then electron-transferred onto a polyvinylidene difluoride membrane (Millipore, USA). Using 1:1000 diluted rabbit anti-mouse or human-CytC (Cell Signaling Technology, CST, USA), -AIF (CST), -EndoG-IgG (Absin, China), -Bid (CST), or -p-Akt (Ser-473, CST) as the primary antibody, and 1:3,000 diluted HRP-conjugated goat anti-rabbit-IgG as the secondary antibody (CST), several western blot assays were performed to detect the target proteins in the mitochondrial or cytosolic fractions. In this study, the β-actin were used as the controls.

### Immunofluorescence assay

J774A.1 or THP-1 cells (1 × 10^5^ per well) were seeded in 12-well culture plates containing 12 × 12 mm coverslip and incubated at 37°C overnight. After washing with PBS, the cell monolayers were infected with *L. interrogans* strain Lai (MOI 100) for 1, 2, 4, or 8 h. Subsequently, cells were loaded with 100 nM MitoTracker Red CMXRos (Invitrogen) for 15 min in culture medium. Then the cells were washed thoroughly with PBS and fixed in 4% paraformaldehyde at 4°C overnight. After washing with PBS, the cells were permeabilized with 0.1% Triton X-100 in PBS for 10 min at room temperature, and then incubated with 5% BSA for 4 h at 4°C. Subsequently, the cells were incubated with 1:500 diluted rabbit anti-mouse or human-AIF- (CST), or -EndoG-IgG (Absin), followed by incubation with 1:1,000 diluted Alexa FluorTM488-conjugated anti-rabbit-IgG (CST) for 1 h at 37°C. After washing with PBS, DAPI (0.1 μg/ml; CST) was added for 5-min incubation at room temperature to stain nuclei. Finally, the nuclear translocation of AIF or EndoG in the leptospire-infected macrophages was observed using a laser confocal microscope (Olympus IX81-FV1000, Japan).

### Quantification of DNA fragmentation

DNA fragmentation assays were performed with a Cellular DNA fragmentation ELISA kit (Roche) according to the manufacturer's protocol (Onaran et al., 2008). Cells were firstly incubated for 2 h at 37°C with 5′-Bromo-2′-deoxy-uridine (BrdU), which can be incorporated into genomic DNA. Subsequently, cells were infected with leptospires for 1, 2, or 4 h. After that, cells were lysed, transferred to a microtiter plate coated with an anti-DNA antibody, and incubated for 1 h at room temperature. After washing with washing solution, anti-BrdU-peroxidase conjugate solution was added, and the plate was incubated for 90 min at room temperature (Onaran et al., 2008). After washing three times, substrate solution was added, and the plate was incubated in the dark on a shaker until color development was sufficient. After stopping the reaction by adding stop solution, a microplate reader (Bio-Rad, Hercules, CA) was used to measure absorbance at 450 nm for each well.

### Detection of intracellular ROS

The intracellular ROS levels in leptospire-infected cells were detected by Dichlorofluorescein diacetate (DCFH-DA) (Sigma), a ROS specific fluorescent dye (Hu et al., 2013). Briefly, the leptospire-infected cells were incubated in medium containing 5 mM DCFH-DA for 30 min at 37°C, and then the fluorescence intensity reflecting ROS levels was detected by laser confocal microscopy (LSM510-Meta, Zeiss, Germany).

### Statistical analysis

Data from at least three independent experiments were averaged to present as mean ± *SD* (standard deviation). Statistical analysis of the differences between the groups was performed using the Student's *t*-test with *P* ≤ 0.05 considered as statistically significant.

## Results

### Macrophage mitochondrion-mediated apoptosis caused by infection with leptospira

**Figure 1:**
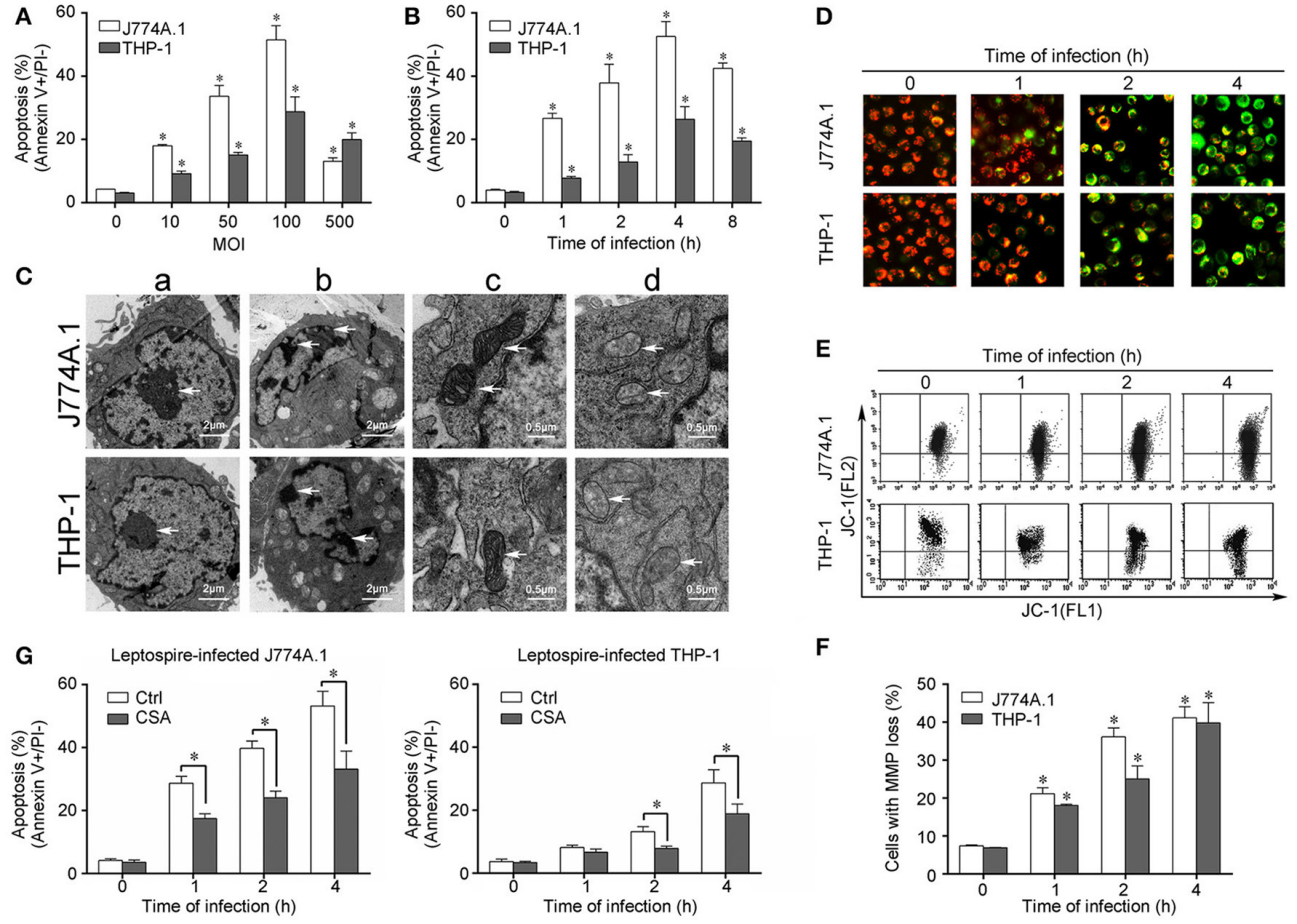
Apoptosis, pathological changes, and MMP decrease in leptospire-infected macrophages. **(A)** Apoptosis of macrophages infected with *L. interrogans* strain Lai at the optimal apoptosis-causing time for different MOIs. J774A.1 and THP-1 cells were infected with leptospires at 4 h. Bars show the mean ± *SD* of three independent experiments. Five thousand cells were analyzed in each specimen. ^*^p < 0.05 vs. apoptotic ratios in leptospire-infected macrophages with an MOI of 10, 50, 100, or 500. **(B)** Apoptosis of macrophages infected with *L. interrogans* strain Lai at the optimal apoptosis-causing MOI for different times. J774A.1 and THP-1 cells were infected at an MOI of 100. Bars show the mean ± *SD* of three independent experiments. Five thousand cells were analyzed in each specimen. ^*^p < 0.05 vs. apoptotic ratios in each macrophage type before infection. **(C)** The representative pathological changes in the nucleus and mitochondria in macrophages infected with *L. interrogans* strain Lai (MOI 100) for 4 h. a: healthly cells showed normal cellular morphology, b: chromatin margination in leptospire-infected macrophages crescent, c: mitochondrial shape in healthly macrophages, d: disappearance of mitochondrial cristae in leptospire-infected macrophages. **(D)** The representative MMP changes in macrophages during infection with *L. interrogans* strain Lai for the indicated times determined by fluorescence microscopy. The red cells have a high MMP while the green cells have a low MMP. **(E)** The representative MMP changes in macrophages during infection with *L. interrogans* strain Lai for the indicated times determined by flow cytometry. The FL2 channel indicates high MMP (red) while the FL1 channel shows low MMP (green). **(F)** Statistical summary of MMP changes by flow cytometry in leptospire-infected macrophages. Statistical data from experiments such as shown in **(E)**. Bars show the mean ± *SD* of three independent experiments. The values at “0” h show the MMP values before infection. Five thousand cells were analyzed in each specimen. ^*^p < 0.05 vs. MMP value of the macrophages before infection. **(G)** CSA blockage of the apoptosis in macrophages infected with *L. interrogans* strain Lai. Bars show the mean ± *SD* of three independent experiments. Five thousand cells were analyzed in each specimen. ^*^p < 0.05 vs. apoptotic ratios in each macrophage type unpretreated with CSA infected with the spirochetes.

Cells of the mouse macrophage cell line J774A.1 and human monocyte cell line THP-1 displayed apoptosis after infection with *L. interrogans* strain Lai. An MOI of 100 caused maximal early apoptosis in both J774A.1 and THP-1 cells (Figure 1A), and when the macrophages were infected with an MOI of 100, apoptosis was maximal at 4 h (Figure 1B). After infection with *L. interrogans* strain Lai at MOI of 100 for 4 h, all the macrophages displayed typical apoptotic morphological changes such as chromatin condensation and margination (Figure 1C,b). Compared to healthy mitochondrial control (Figure 1Cc), the cristae of mitochondria in the leptospire-infected macrophages had disappeared (Figure 1Cd). In addition, JC-1-based fluorescence microscopy and flow cytometry showed a gradual but significant decrease of MMP in both of leptospire-infected macrophag cell lines (Figures 1D–F). Pretreatment of the macrophages with CSA, a MPT blocker, significantly decreased the leptospire-induced apoptotic ratios (Figure 1G). These results suggested that *Leptospira* infection causes mitochondrial changes in mouse and human macrophages, which is consistent with mitochondrion-mediated apoptosis.

### Caspase-8 but not caspase-9/CytC involved in leptospire-induced apoptosis

**Figure 2:**
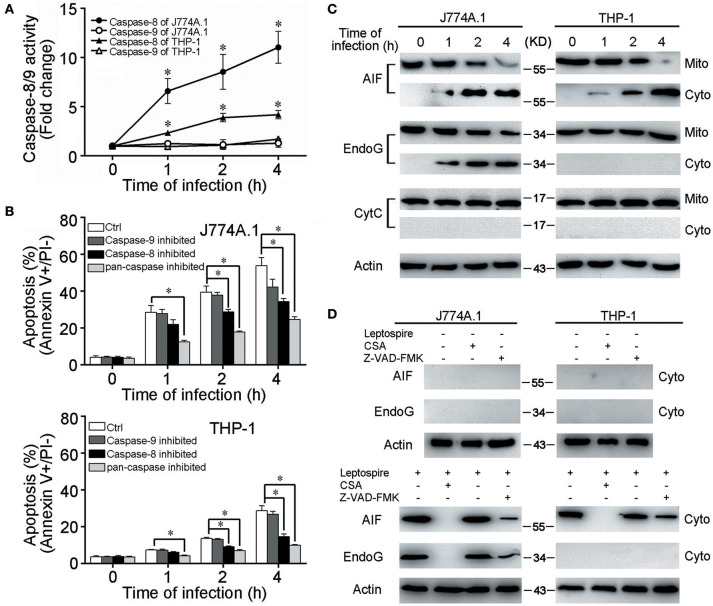
Caspase-8/9 activation and mitochondrial release of AIF and EndoG in leptospire-infected macrophages. **(A)** Caspase activation during *L. interrogans* strain Lai infection. Macrophages were incubated with leptospires at an MOI of 100 for the indicated times. Cell lysates were prepared, and the activities of caspase were determined with the fluorogenic substrates Ac-IETD-AFC (for caspase-8), and Ac-LEHD-AFC (for caspase-9). Bars show the mean ± SD of three independent experiments. ^*^p < 0.05 vs. caspase activation in each macrophage type before infection. The value for the uninfected control was set at one-fold. **(B)** Apoptosis of macrophages pretreated with caspase-8, 9 or pan-caspase inhibitor infected with *L. interrogans* strain Lai. Cells were pretreated with Z-IETD-FMK, Z-LEHD-FMK, Z-VAD-FMK to inhibit the activation of caspase-8, caspase-9 or pan-caspase. Bars show the mean ± *SD* of three independent experiments. ^*^p < 0.05 vs. apoptotic ratios in each macrophage type infected with the spirochete for 1, 2, or 4 h but unpretreated with inhibitor. **(C)** The release of AIF, EndoG and CytC from mitochondria in the macrophages during infection with leptospires for the indicated times. **(D)** CSA or Z-VAD-FMK blockage of AIF and EndoG release from mitochondria. Macrophages were incubated with or without leptospires at an MOI of 100 for 4 h. Representative results of three experiments with consistent results are shown.

CytC release from mitochondria and subsequent caspase-9 activation are necessary steps to induce apoptosis through the mitochondrion-related caspase-dependent pathway (Green and Kroemer, 2004). However, in all the macrophages infected with *L. interrogans* strain Lai, only caspase-8 was activated and caspase-9 was not (Figure 2A). When the macrophages were pretreated with Z-VAD-FMK (pan-caspase inhibitor) or Z-IETD-FMK (caspase-8 inhibitor), the apoptotic ratios of leptospire-infected macrophages were substantially decreased, while no significant decrease in apoptotic ratios were observed in Z-LEHD-FMK (caspase-9 inhitor) pretreated cells (Figure 2B). Furthermore, no mitochondrial release of CytC from the infected macrophages was detected (Figure 2C). These data suggested that the typical CytC-caspase-9 pathway is not involved in mitochondrion-related apoptosis of macrophages caused by infection with *Leptospira*.

### Mitochondrial release of AIF and EndoG in leptospire-infected macrophages

AIF and EndoG have been shown to mediate mitochondrion-related apoptosis when they are released from mitochondria (Li et al., 2001; Cregan et al., 2004; Green and Kroemer, 2004). In leptospire-infected J774A.1 cells, both AIF and EndoG were released from mitochondria into the cytosol, whereas in infected THP-1 cells, only the mitochondrial release of AIF was detectable (Figure 2C). When the macrophages were pretreated with CSA, the mitochondrial release of both AIF and EndoG was completely inhibited, while pretreatment with Z-VAD-FMK only decreased mitochondrial release of AIF and EndoG (Figure 2D). These results indicated that infection with leptospire induces mouse and human macrophages to selectively release AIF and/or EndoG from mitochondria, and that AIF and/or EndoG are involved in *Leptospira*-induced mitochondrion-related macrophage apoptosis in both caspase-dependent and caspase-independent ways.

### Nuclear translocation of AIF and EndoG and DNA fragmentation during apoptosis

**Figure 3:**
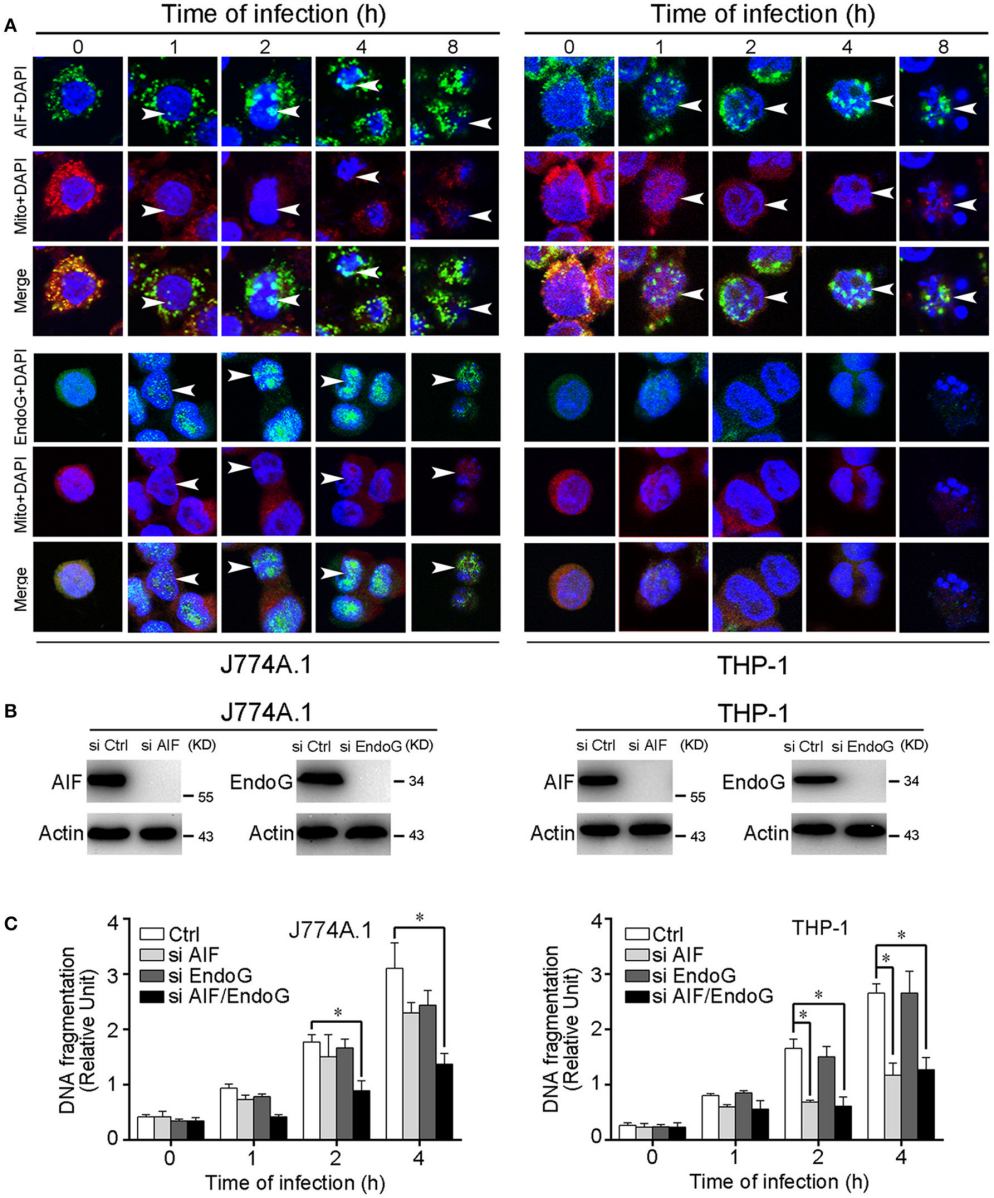
Nuclear translocation of AIF, EndoG, and the DNA fragmentation in leptospire-infected macrophages. **(A)** The representative nuclear translocation of AIF and Endo-G in J774A.1 and THP-1 cells infected with *L. interrogans* strain Lai for the indicated times. The small green points indicate AIF or EndoG protein stained with FITC-conjugated antibody. The red points indicate the mitochondria stained with MitoTracker Red CMXRos. The blue spots indicate nuclei stained with DAPI. Arrows: AIF or EndoG nuclei translocation. **(B)** The expression of AIF and EndoG in siRNA pretreated J774A.1 and THP-1 cells. Macrophages were transfected with Ctrl siRNA, si AIF or si EndoG, and then infected with leptospires for 4 h. **(C)** DNA fragmentation in siRNA pretreated J774A.1 and THP-1 cells infected with *L. interrogans* strain Lai. Cells pretreated with Ctrl siRNA, si AIF, si EndoG, or both si AIF and si EndoG were infected with leptospires for the indicated times. Bars show the mean ± *SD* of three independent experiments. ^*^p < 0.05 for comparison with the cells transfected with Ctrl siRNA. All data are representative of three independent experiments.

Previous studies showed that AIF and EndoG that are released from mitochondria into the cytosol further translocate into the nucleus, resulting in chromatin condensation and DNA fragmentation (Li et al., 2001; Hong et al., 2004). In the leptospire-infected macrophages, AIF and/or EndoG proteins released from mitochondria into the cytosol were also translocated into the nucleus (Figure 3A). In addition, nuclear DNA fragmentation was detected by using a cellular DNA fragmentation ELISA. We subsequently used AIF RNAi (si AIF), EndoG RNAi (si EndoG), or a combination of both AIF and EndoG RNAi (si AIF/EndoG) to decrease the expression of AIF and EndoG in macrophages (Figure 3B). We found that knockdown of either AIF or EndoG alone could not efficiently decrease DNA fragmentation in J774A.1 cells infected with leptospire, while AIF and EndoG double knockdown substantially decreased DNA fragmentation (Figure 3C). However, since there is no EndoG released from mitochondria, knockdown of AIF alone could efficiently decrease DNA fragmentation in THP-1 cells (Figure 3C). These data demonstrated that the release of mitochondrial AIF and/or EndoG induces DNA fragmentation, which is required for leptospire to induce apoptosis during an infection.

### Activated bid causes the release of AIF and EndoG during apoptosis

**Figure 4:**
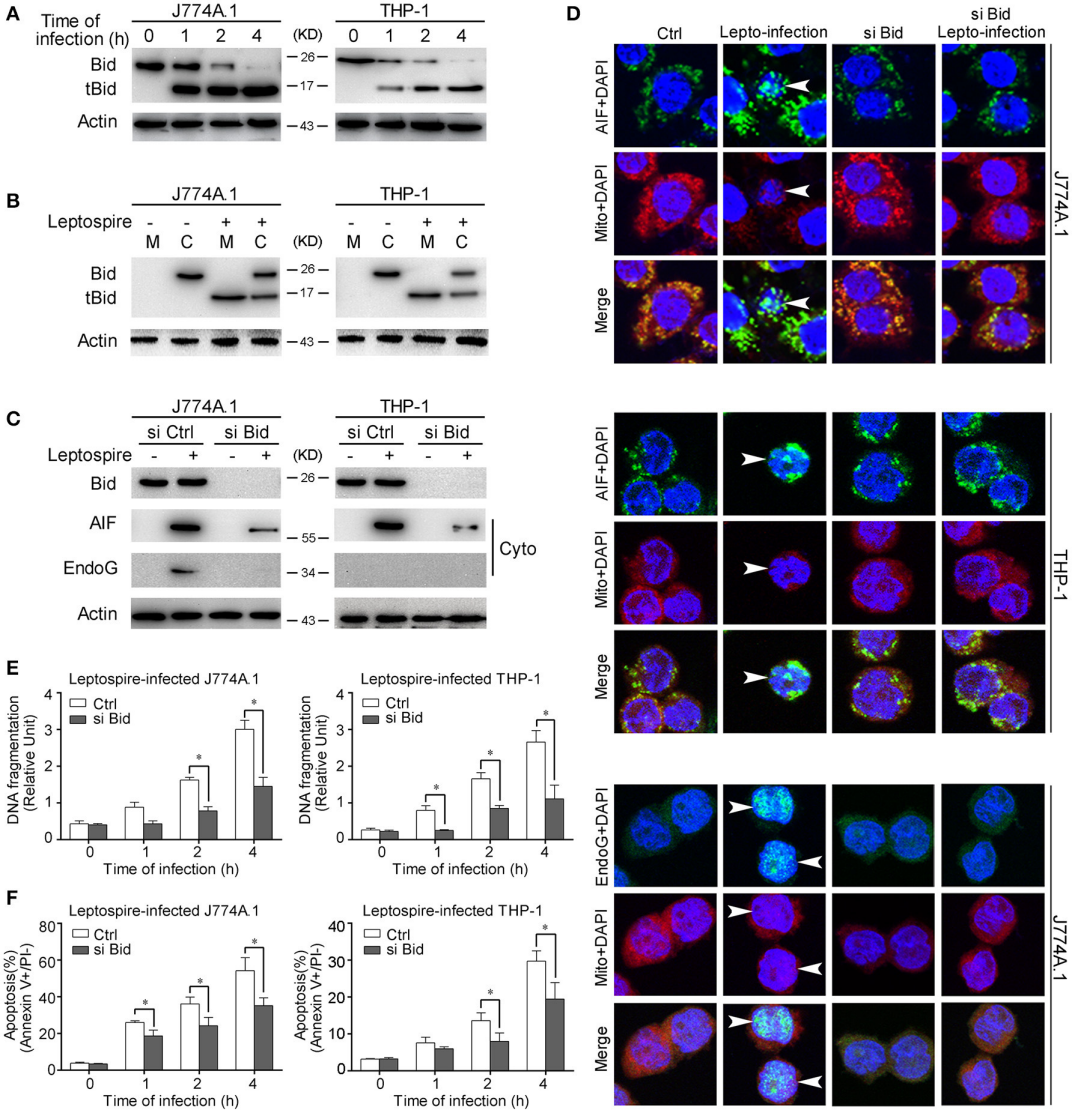
Bid activation mediates the release and nuclear translocation of AIF, EndoG, and the subsequent DNA fragmentation and apoptosis. **(A)** The activation of Bid in the macrophages infected with leptospires at the indicated times. **(B)** The subcellular location of Bid and tBid during infection. Macrophages were infected with leptospires for 4 h and subjected to subcellular fractionation. The mitochondrial (M) or cytosolic (C) fractions were immunoblotted for Bid and tBid. **(C)** The reduction of the release of AIF and EndoG from mitochondria in si Bid pretreated macrophages during the infection. Macrophages were transfected with si Bid or Ctrl siRNA, and then infected with leptospires for 4 h. **(D)** The reduction of the nuclear translocation of AIF and EndoG in si Bid pretreated macrophages during the infection. Macrophages were transfected with si Bid or Ctrl siRNA, and then infected with leptospires for 4 h. Arrows: AIF or EndoG nuclei translocation. **(E)** The reduction of the DNA fragmentation in si Bid pretreated macrophages during the infection. Cells pretreated with Ctrl siRNA or si Bid were infected with leptospires for the indicated times. Bars show the mean ± SD of three independent experiments. ^*^p < 0.05 for comparison with the Ctrl siRNA. **(F)** The reduction of the apoptosis in si Bid pretreated macrophages during the infection. Cells pretreated with Ctrl siRNA or si Bid were infected with leptospires for the indicated times. Bars show the mean ± SD of three independent experiments. ^*^p < 0.05 for comparison with the Ctrl siRNA. Representative results of three experiments with consistent results are shown.

Bid is a pro-apoptotic member of the Bcl-2 family. Previous studies have demonstrated that the activated Bid can mediate the release of AIF and EndoG (Li et al., 2001; Landshamer et al., 2008; Guo et al., 2015). Our results confirmed that Bid was cleaved into tBid, the active truncated form of Bid, during the time course of leptospire infection (Figure 4A). Simultaneously, tBid translocated from the cytosol into the mitochondria, which induced the release of mitochondrial proteins (Figure 4B). When the expression of Bid was knocked down using siRNA, the release and nuclear translocation of AIF and/or EndoG was affected (Figures 4C,D). In addition, Bid knockdown also substantially decreased nuclear DNA fragmentation and apoptosis in macrophages infected with leptospire (Figures 4E,F). These results demonstrate that Bid is able to induce the release of AIF and/or EndoG and consequently apoptosis.

### Caspase-8 induces the activation of bid and the release of AIF and EndoG during the apoptosis

**Figure 5:**
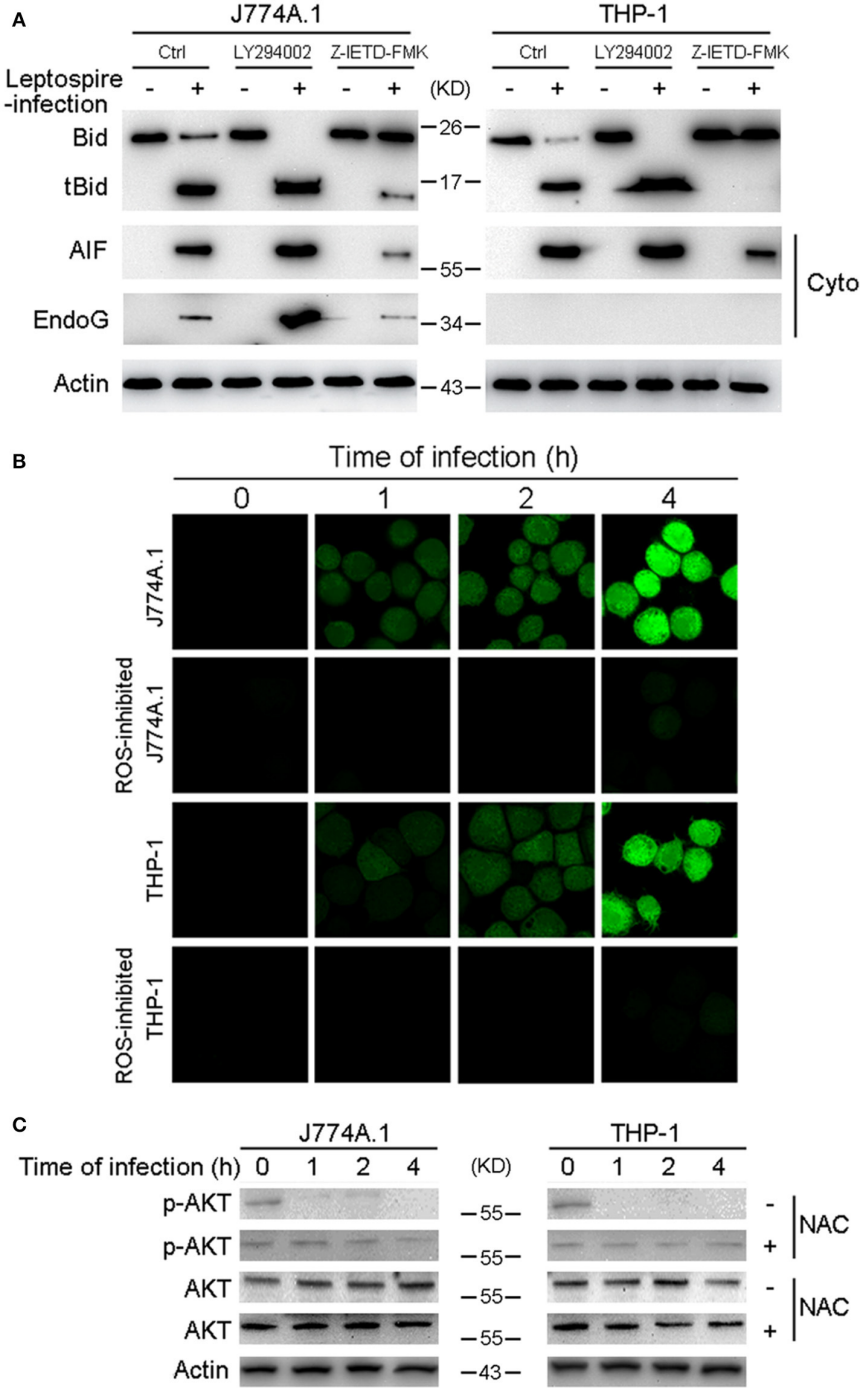
Caspase-8 activation and ROS induced Akt inactivation mediates the activation of Bid and the release of AIF, EndoG. **(A)** The activation of Bid and the release of AIF and EndoG in Caspase-8 or Akt inhibited macrophages during infection. The macrophages pretreated with caspase-8 inhibitor Z-IETD-FMK or PI3K/Akt pathway inhibitor LY294002 were infected with leptospires for 4 h. **(B)** ROS level in macrophages infected with *L. interrogans* strain Lai for the indicated times. The cells were stained with DCFH-DA, an ROS-specific fluorescent dye, which become green due to an increase in ROS levels. NAC was used to neutralize intracellular ROS. **(C)** The dephosphorylation of Akt in macrophages infected with *L. interrogans* strain Lai for the indicated times. NAC was used to neutralize intracellular ROS. Representative results of three experiments with consistent results are shown.

Bid usually remains in an inactive form in the cytosolic fraction of living cells and is cleaved and activated by caspase-8 in response to TNF alpha or Fas ligand (Kantari and Walczak, 2011). Our data have already demonstrated that caspase-8 was activated during leptospire-induced apoptosis (Figure 2A). Subsequently, Z-IETD-FMK was used to inactivate caspase-8, which efficiently decreased activation of Bid and the release of AIF or EndoG (Figure 5A). These results indicate that activated caspase-8 mediates the cleavage and activation of Bid and the release of AIF and/or EndoG.

### ROS-induced akt inactivation mediates bid-induced release of AIF during the apoptosis

Akt activation is able to inhibit Bid processing and Bid-induced apoptosis (Majewski et al., 2004; Goncharenko-Khaider et al., 2010). Moreover, our previous study showed that phosphorylated (activated) Akt (p-Akt) was decreased during the time course of an infection, and that leptospire-induced apoptosis through the Akt-inactivation pathway in macrophages (Hu et al., 2013). Thus, we speculated that Akt is the upstream apoptotic regulator in Bid-induced apoptosis. Our data revealed that LY294002, an Akt inhibitor, efficiently increased the activation of Bid and the release of AIF and/or EndoG (Figure 5A). These results indicate that Akt inactivation mediates Bid-induced downstream apoptotic events instigated by leptospire-infection. It has been reported that ROS play critical roles in the regulation of diverse functional pathways involved in proliferation, apoptosis, and cellular transformation (Simon et al., 2000; Blomgran et al., 2007). The ROS specific fluorescent staining assay showed that ROS levels were rapidly increased in J774A.1 and THP-1 cells after infection with leptospire, and this increase could be significantly blocked by anti-oxidant NAC (Figure 5B). Furthermore, the decreased p-Akt protein levels observed after leptospire-infection was reversed after the removal of intracellular ROS by NAC, while the level of total Akt was not changed (Figure 5C). These results indicate that ROS induced Akt inactivation mediates Bid-induced release of AIF and EndoG during apoptosis.

## Discussion

Host-pathogen interactions play an important role in the pathogenesis of infectious diseases (Haraga et al., 2008; Behar et al., 2011; Medzhitov et al., 2012). Macrophages play a crucial role in the innate immune response to eliminate invading microbes by phagocytosis. When fulfilling this function, macrophages are controlled via processes regulating both cell proliferation and apoptosis. At the same time, pathogens utilize idiosyncratic mechanisms to modulate the apoptotic pathways and that the outcomes of this modulation vary between different pathogens (Rosenberger and Finlay, 2003; Rudel et al., 2010; Behar et al., 2011). In leptospirosis patients, macrophages, but not neutrophils, are essential for killing of the phagocytosed leptospires (Chierakul et al., 2004; Davis et al., 2009; Chen et al., 2017). Therefore, macrophages play a key defensive role in the host during leptospirosis.

Two core pathways exist to induce apoptosis, the intrinsic-mitochondrial pathway, and extrinsic-death receptor pathway (Ouyang et al., 2012). Mitochondria play a key role in intrinsic cell death signaling by both caspase-dependent and caspase-independent pathways (Green and Kroemer, 2004). Our previous studies showed that the mitochondria in leptospire-infected J774A.1 cells are morphologically disrupted and that AIF and/or EndoG could be released from mitochondrion during leptospire-infection (Li et al., 2007; Hu et al., 2013).

In the present study, we demonstrated mitochondrial injury (disappearance of cristae and decreased MMP) in mouse J774A.1 macrophages and human PMA-induced THP-1 macrophages during infection with *L. interrogans* strain Lai, and the MPT blocker CSA caused a decrease in apoptotic ratios (Figure 1). However, no activation of caspase-9 and mitochondrial release of CytC was observed during an infection, while AIF and/or EndoG showed translocation from the mitochondria to the cytosol (Figure 2). Moreover, in the leptospire-infected macrophages, the caspase-8 inhibitor Z-IETD-FMK and pan-caspase inhibitor Z-VAD-FMK partly blocked apoptosis, while no significant decrease in apoptotic ratios were observed in Z-LEHD-FMK (caspase-9 inhibitor) pretreated cells (Figure 2). These data indicated that mitochondrion-related caspase-9-CytC-independent pathways signaling through mitochondrial AIF and/or EndoG are involved in leptospire-induced apoptosis.

In the apoptotic pathway mediated by EndoG, AIF, and Smac, their release from mitochondria into the cytosol and subsequent translocation into the nucleus are essential steps to stimulate apoptosis (Li et al., 2001; Vega-Manriquez et al., 2007; Ashktorab et al., 2008). AIF binds to chromosomal DNA to promote chromatin condensation, EndoG is an endogenous restriction endonuclease, and Smac induces apoptosis by suppressing the apoptotic inhibitor of caspase-3 protein (Li et al., 2001; Cande et al., 2004; Rudy et al., 2008). In the present study, we observed that AIF and EndoG were released from mitochondria and translocated into the nucleus in the leptospire-infected mouse macrophage cell line J774A.1, while in the infected human macrophage cell line THP-1, only the mitochondrial release and nuclear translocation of AIF was detectable (Figure 2C, 3A). Smac was not released from mitochondria during leptospire-induced macrophage apoptosis (data not shown). The released and translocated AIF and EndoG subsequently caused DNA fragmentation in the leptospire-infected macrophages (Figures 3B,C). In previous studies, AIF and EndoG were shown to mediate caspase-independent apoptosis of macrophages infected with *M. tuberculosis* or *H. pylori* (Vega-Manriquez et al., 2007; Ashktorab et al., 2008), while Smac was frequently found to play a role in the apoptosis of virus-infected non-phagocytic cells (Kominsky et al., 2002; Mocarski et al., 2011). These data demonstrate the diversity of mitochondria-related caspase-independent apoptotic signaling mechanisms in different host cells stimulated by different pathogens. Based on these findings, we demonstrate that a leptospire infection causes the release and translocation of AIF and/or EnodG, which induces DNA fragmentation that is associated with apoptosis.

The extrinsic pathway is triggered by binding of Fas plasma-membrane death receptor with its extracellular ligand FasL. The Fas/FasL composite recruits death domain-containing protein (FADD) and pro-caspase-8, which aggregate to become the death-inducing signaling complex (Lamkanfi and Dixit, 2010). Subsequently, this protein complex activates pro-caspase-8, which proceeds to trigger pro-caspase-3, the penultimate enzyme for the execution of the apoptotic process (Yang et al., 2015). Our previous studies demonstrated that FADD-caspase-8,−3-dependent pathways were activated during apoptosis of macrophages infected with *L. interrogans* strain Lai (Jin et al., 2009; Hu et al., 2013), however, the link between caspase-8 and mitochondrial apoptosis remained unknown. BID is a pro-apoptotic member of the Bcl 2 family, which serves as a direct molecular link between caspase-8 activation and the mitochondrial death machinery (Kantari and Walczak, 2011). After the inactive form of Bid in the cytosolic fraction is cleaved and activated by caspase-8 in response to FasL, the C-terminal fragment of BID (tBid) translocates onto mitochondria, which is sufficient to trigger AIF and EndoG release, resulting in cell apoptosis (Kaufmann et al., 2012; Guo et al., 2015). In the present study, we found that Bid was cleaved into tBid and the latter translocated onto the mitochondria during the infection (Figures 4A,B). When Bid was knocked down by siRNA, the release and nuclear translocation of AIF and EndoG, the ratio of DNA fragmentation and apoptosis were significant decreased during the infection (Figures 4C–F). These data indicate that Bid was activated during the infection and induced the release of AIF and EndoG, which resulted in apoptosis. In addition, we also found that caspase-8 was activated during the infection (Figure 2A), and in the caspase-8 inhibited macrophages, the ratio of apoptosis (Figure 2B), the cleavage of Bid, and the release of AIF and EndoG from mitochondria were significantly decreased (Figure 5A). These data indicated that caspase-8 induces the activation of Bid, which controls the release of AIF and EndoG during leptospire-induced apoptosis in macrophages.

Since the pan-caspase inhibitor Z-VAD-FMK only decreased but not blocked the mitochondrial release of AIF and EndoG (Figure 2D), and the caspase-8 inhibitor Z-IETD-FMK also just decreased the activation of Bid (Figure 5A), we postulated that there might be other upstream regulators besides caspase-8 to activate the Bid-AIF/EndoG pathway. Macrophages are professional phagocytes, which participate in the primary host defense against invading pathogens (DeLeo, 2004). Upon contact with pathogens and during phagocytosis, macrophages generate ROS, which function in conjunction with several granule components to constitute an effective system for killing invading pathogens (Martinon, 2010; Dupre-Crochet et al., 2013). ROS are important component in the complex modulation of macrophage apoptosis (Simon et al., 2000). The serine/threonine kinase Akt plays a critical role in controlling the survival and apoptosis of mammalian cells. It has been reported that ROS-driven Akt dephosphorylation at Ser-473 is involved in apoptosis (Inoue et al., 2004; Cao et al., 2009), and Akt inactivation mediated Bid-induced cell apoptosis (Guo et al., 2015). In our previous study, we found that leptospires induced an intrinsic mode of apoptosis in macrophages, which is depended on intracellular ROS as upstream initiators (Hu et al., 2013). However, the mechanism of ROS-induced apoptosis during infection was still unknown. In the present study, ROS scavengers effectively inhibited leptospire-infection induced increases of dephosphorylated Akt in macrophages, indicating that ROS signaling is upstream of the Akt pathway (Figures 5B,C). Next, we found that in the macrophages pretreated with the PI3K/Akt inhibitor LY294002, which enhances Akt inactivation, the cleavage of Bid and the release of AIF and EndoG were increased (Figure 5A). These data suggested that ROS induced inactivate Akt during an infection, which controls the cleavage of Bid and subsequent release of AIF and EndoG.

**Figure 6:**
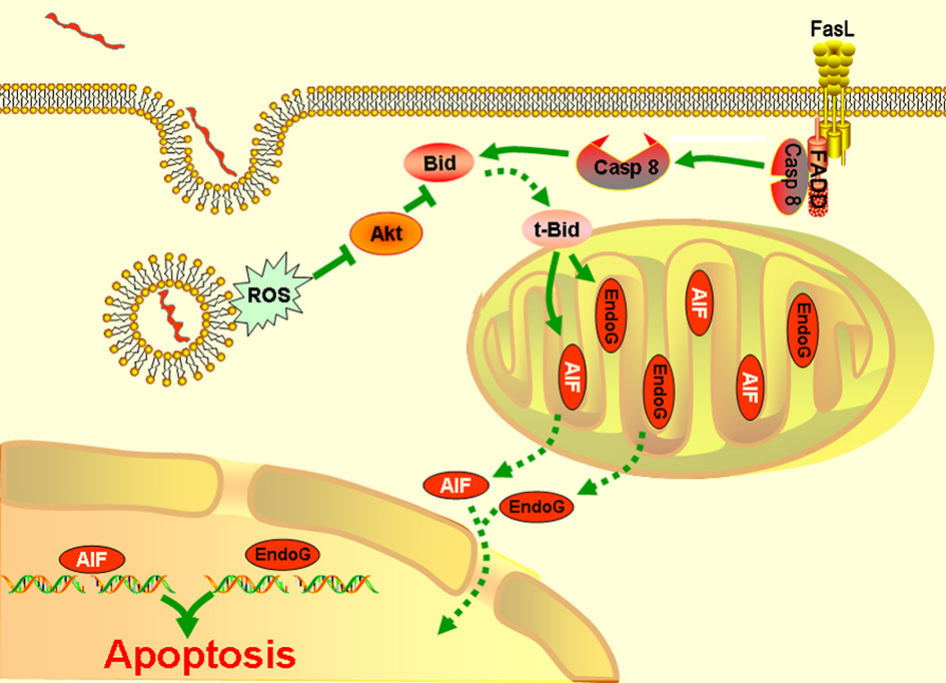
A model of leptospire-induced apoptosis in macrophages through bid-induced release of AIF/ Endog signaling pathway. Upon contact with leptospires and during phagocytosis, macrophages generate ROS. ROS triggers the dephosphorylation of Akt. The decrease of p-Akt activated Bid to be cleaved into tBid, which reduced the mitochondrial membrane permeabilization, leading to release of AIF and EndoG. AIF and EdnoG translocated into the nuclear and caused the DNA fragmentation and apoptosis. In parallel, infection induced the activation of caspase-8 through Fas/FasL-FADD pathway. Caspase-8 also cleaved the Bid into tBid, which caused the release and nuclear translocation of AIF and EndoG, and induced the apoptosis. The green solid line: stimulatory or inhibitory modification; the green dotted line: translocation.

Taken together, the results presented in our study showed that AIF and/or EndoG mediate the mitochondrion-related apoptosis of macrophages during infection with *Leptospira* species. This process is modulated by Bid, which is activated by both caspase-8 and the ROS-Akt signal pathway (Figure 6).

## Author contributions

Designed the experiments: W-LH, H-YD, S-JL, and JY. Performed the experiments: W-LH, H-YD, and YL. Analyzed the data: W-LH, H-YD, and JY. Contributed reagents/materials/analysis tools: YL, DO. Wrote the paper: W-LH, H-YD, and JY.

### Conflict of interest statement

The authors declare that the research was conducted in the absence of any commercial or financial relationships that could be construed as a potential conflict of interest.
