# Correction: Bid-Induced Release of AIF/EndoG from Mitochondria Causes Apoptosis of Macrophages during Infection with *Leptospira interrogans*

**DOI:** 10.3389/fcimb.2025.1635286

**Published:** 2025-07-15

**Authors:** Wei-Lin Hu, Hai-Yan Dong, Yang Li, David M. Ojcius, Shi-Jun Li, Jie Yan

**Affiliations:** ^1^ Department of Medical Microbiology and Parasitology, Zhejiang University School of Medicine, Hangzhou, China; ^2^ Division of Basic Medical Microbiology, State Key Laboratory for Diagnosis and Treatment of Infectious Diseases, The First Affiliated Hospital, Zhejiang University School of Medicine, Hangzhou, China; ^3^ Department of Medical Microbiology and Immunology, Wenzhou Medical University, Wenzhou, China; ^4^ Department of Biomedical Sciences, University of the Pacific, Arthur Dugoni School of Dentistry, San Francisco, CA, United States; ^5^ Institute of Communicable Disease Control and Prevention, Guizhou Provincial Centre for Disease Control and Prevention, Guiyang, China

**Keywords:** apoptosis, *Leptospira*, Bid, AIF, EndoG, macrophage

In the published article, there was an error in **Figure 1E** as published. The representative flow dot plots were misplaced. The corrected **Figure 1** appears below.

**Figure 1:**
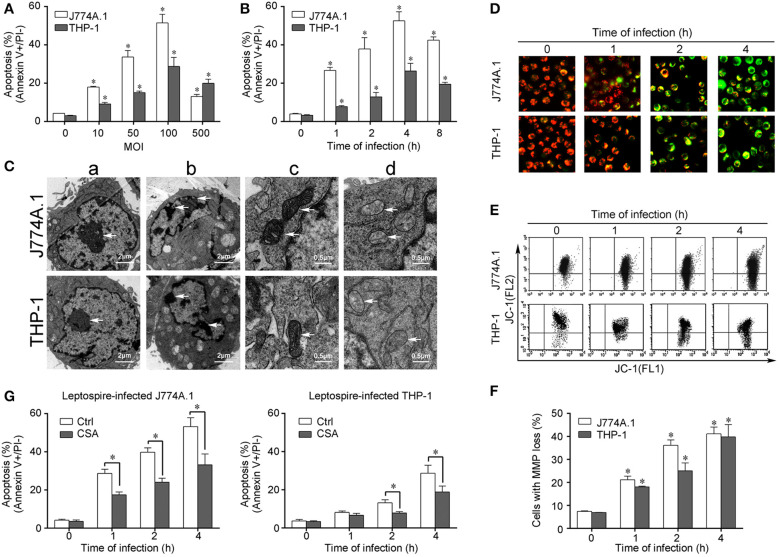
Apoptosis, pathological changes, and MMP decrease in leptospire-infected macrophages. **(A)** Apoptosis of macrophages infected with *L. interrogans* strain Lai at the optimal apoptosis-causing time for different MOIs. J774A.1 and THP-1 cells were infected with leptospires at 4 h. Bars show the mean ± *SD* of three independent experiments. Five thousand cells were analyzed in each specimen. *p < 0.05 vs. apoptotic ratios in leptospire-infected macrophages with an MOI of 10, 50, 100, or 500. **(B)** Apoptosis of macrophages infected with *L. interrogans* strain Lai at the optimal apoptosis-causing MOI for different times. J774A.1 and THP-1 cells were infected at an MOI of 100. Bars show the mean ± *SD* of three independent experiments. Five thousand cells were analyzed in each specimen. *p < 0.05 vs. apoptotic ratios in each macrophage type before infection. **(C)** The representative pathological changes in the nucleus and mitochondria in macrophages infected with *L. interrogans* strain Lai (MOI 100) for 4 h. a: healthly cells showed normal cellular morphology, b: chromatin margination in leptospire-infected macrophages crescent, c: mitochondrial shape in healthly macrophages, d: disappearance of mitochondrial cristae in leptospire-infected macrophages. **(D)** The representative MMP changes in macrophages during infection with *L. interrogans* strain Lai for the indicated times determined by fluorescence microscopy. The red cells have a high MMP while the green cells have a low MMP. **(E)** The representative MMP changes in macrophages during infection with *L. interrogans* strain Lai for the indicated times determined by flow cytometry. The FL2 channel indicates high MMP (red) while the FL1 channel shows low MMP (green). **(F)** Statistical summary of MMP changes by flow cytometry in leptospire-infected macrophages. Statistical data from experiments such as shown in **(E)**. Bars show the mean ± *SD* of three independent experiments. The values at “0” h show the MMP values before infection. Five thousand cells were analyzed in each specimen. *p < 0.05 vs. MMP value of the macrophages before infection. **(G)** CSA blockage of the apoptosis in macrophages infected with *L. interrogans* strain Lai. Bars show the mean ± *SD* of three independent experiments. Five thousand cells were analyzed in each specimen. *p < 0.05 vs. apoptotic ratios in each macrophage type unpretreated with CSA infected with the spirochetes.

The authors apologize for this error and state that this does not change the scientific conclusions of the article in any way. The original article has been updated.

